# HyScreen: A Ground-Based Imaging System for High-Resolution Red and Far-Red Solar-Induced Chlorophyll Fluorescence

**DOI:** 10.3390/s22239443

**Published:** 2022-12-02

**Authors:** Huaiyue Peng, Maria Pilar Cendrero-Mateo, Juliane Bendig, Bastian Siegmann, Kelvin Acebron, Caspar Kneer, Kari Kataja, Onno Muller, Uwe Rascher

**Affiliations:** 1 Institute of Bio- and Geosciences, IBG-2: Plant Sciences, Forschungszentrum Jülich GmbH, 52428 Jülich, Germany; 2 Laboratory of Earth Observation, Image Processing Laboratory, University of Valencia, 46980 Paterna, Spain; 3 Specim Spectral Imaging Ltd., 90590 Oulu, Finland

**Keywords:** imaging spectroscopy, proximal sensing, hyperspectral, calibration, empirical line method, red SIF

## Abstract

Solar-induced chlorophyll fluorescence (SIF) is used as a proxy of photosynthetic efficiency. However, interpreting top-of-canopy (TOC) SIF in relation to photosynthesis remains challenging due to the distortion introduced by the canopy’s structural effects (i.e., fluorescence re-absorption, sunlit-shaded leaves, etc.) and sun–canopy–sensor geometry (i.e., direct radiation infilling). Therefore, ground-based, high-spatial-resolution data sets are needed to characterize the described effects and to be able to downscale TOC SIF to the leafs where the photosynthetic processes are taking place. We herein introduce HyScreen, a ground-based push-broom hyperspectral imaging system designed to measure red ($F_{687}$) and far-red ($F_{760}$) SIF and vegetation indices from TOC with single-leaf spatial resolution. This paper presents measurement protocols, the data processing chain and a case study of SIF retrieval. Raw data from two imaging sensors were processed to top-of-canopy radiance by dark-current correction, radiometric calibration, and empirical line correction. In the next step, the improved Fraunhofer line descrimination (iFLD) and spectral-fitting method (SFM) were used for SIF retrieval, and vegetation indices were calculated. With the developed protocol and data processing chain, we estimated a signal-to-noise ratio (SNR) between 50 and 200 from reference panels with reflectance from 5% to 95% and noise equivalent radiance (NER) of 0.04 (5%) to 0.18 (95%) mW m${}^{-2}$ sr${}^{-1}$ nm${}^{-1}$. The results from the case study showed that non-vegetation targets had SIF values close to 0 mW m${}^{-2}$ sr${}^{-1}$ nm${}^{-1}$, whereas vegetation targets had a mean $F_{687}$ of 1.13 and $F_{760}$ of 1.96 mW m${}^{-2}$ sr${}^{-1}$ nm${}^{-1}$ from the SFM method. HyScreen showed good performance for SIF retrievals at both $F_{687}$ and $F_{760}$; nevertheless, we recommend further adaptations to correct for the effects of noise, varying illumination and sensor optics. In conclusion, due to its high spatial resolution, Hyscreen is a promising tool for investigating the relationship between leafs and TOC SIF as well as their relationship with plants’ photosynthetic capacity.

## 1. Introduction

In times of global climate change, quantifying photosynthetic traits efficiently and non-invasively is a key to better understanding the spatio–temporal adaptation of plants’ primary metabolism and to thus improve the early detection of stress in order to sustainably manage plant production [1,2]. To estimate plants’ photosynthesis, chlorophyll fluorescence (ChlF) has been widely used because of its direct connection with the dynamic regulation of photosynthesis at the photosystem level. When chlorophyll molecules are excited by absorbed radiant fluxes, re-emitted fluorescence photons compete with photochemical quenching and non-photochemical quenching energy dissipation (NPQ). These three de-excitation processes are tightly interrelated and are also constantly adjusted under changing environmental conditions and stress levels [3]. The ChlF emission of plants covers the spectral range from 650–800 nm, which is characterized by two peaks, one in the red region (around 685 nm) and the other in the far-red spectral region (around 740 nm), where Photosystem I (PSI) mainly emits ChlF in the far-red range, and Photosystem II (PSII) emits ChlF both in the red and far-red ranges [4].

Solar-induced chlorophyll fluorescence (SIF) can serve as a real-time proxy of photosynthesis under natural illumination and can be applied on a larger scale in contrast to active fluorescence measurement techniques [3,5]. However, measuring SIF is challenging, as the relatively small signal is superimposed on the reflected radiance of plants [6]. Therefore, SIF is commonly retrieved by taking advantage of solar or the Earth’s atmospheric absorption bands where irradiance transmission through the atmosphere is low [7,8]. The typically used spectral bands for SIF retrieval are the solar Fraunhofer lines (Fe (758.8 nm) and KI (770.1 nm) [9]) or Earth’s atmosphere telluric oxygen absorption bands ($O_{2}B$ (687–692 nm) and $O_{2}A$ (759–770 nm)) because their strongest absorption features at 687 nm and 760 nm are close to the SIF emission peaks. Therefore, red ($F_{687}$) and far-red ($F_{760}$) emitted fluorescence can be retrieved at these two oxygen absorption bands. Since SIF only represents a fraction of the radiance measured by a sensor, high signal-to-noise ratio (SNR) as well as adequate measurement protocols and sensor calibration are required for reliable SIF retrieval [8,10,11].

SIF can be sensed from ground-based instruments up to satellites, but there is still a gap in understanding the downscaling of top-of-canopy (TOC) SIF to leaf-level SIF [6,12]. Canopy architecture influences how leaf-emitted SIF is scattered and re-absorbed in the canopy and how absorbed photosynthetically active radiation (PAR) changes within the canopy [13,14]. Furthermore, viewing geometry influences measured SIF [15]. Physically based radiative transfer models can help with understanding these processes, but measured data are mandatory to validate modeled results or as input data to parameterize models [16]. At the landscape level, airborne imaging spectrometers (e.g., HyPlant) [12,17] and unmanned aerial vehicles (UAVs) equipped with point spectrometers [18,19] are essential for bridging the SIF measurement from TOC to satellite. For the scaling gap between leaf and TOC, existing proximal point spectroradiometer systems such as FloX [20] have limited suitability when investigating canopy structures, as the signal is always an integration of the field of view of the sensor. Additional information, such as fractional vegetation cover, sunlit and shaded parts of the canopy, and leaf angles and orientations, can only be investigated with imaging sensors. Some studies have successfully retrieved $F_{760}$ from imaging systems [14,21,22,23]. Their measurements showed spatial and temporal variation of SIF responding to herbicide stress, canopy structure and absorbed photosynthetically active radiation (APAR), corroborating the need of SIF high-spatial-resolution imagery to overcome the challenge of linking TOC fluorescence with leaf-level photosynthetic efficiency, as listed in [4]. However, ground measurements of red SIF ($F_{687}$) have been challenging due to limited spectral resolutions and signal-to-noise ratios of previous imaging spectrometers. Nevertheless, to characterize the contributions of PSII and PSI to the total ChlF emission and, consequently, to understand the dynamics of ChlF and photosynthesis, the measurement of both $F_{687}$ and $F_{760}$ is mandatory [24,25,26].

In this study, we introduce the HyScreen imaging spectrometer system, which allows the retrieval of red and far-red fluorescence at close canopy where single leaves can be spatially resolved. HyScreen consists of two high-spatial- and high-spectral-resolution imaging spectrometers. One allows the retrieval of $F_{760}$ and $F_{687}$, and the other is used for calculating vegetation indices. This paper presents a detailed technical description of HyScreen, introduces the data acquisition protocol and gives insights into the developed processing chain. A case study with structurally simple samples is presented to illustrate the performance of HyScreen. Finally, measurement uncertainties are discussed.

## 2. Materials and Methods

### 2.1. Hyperspectral Sensors

**Figure 1:**
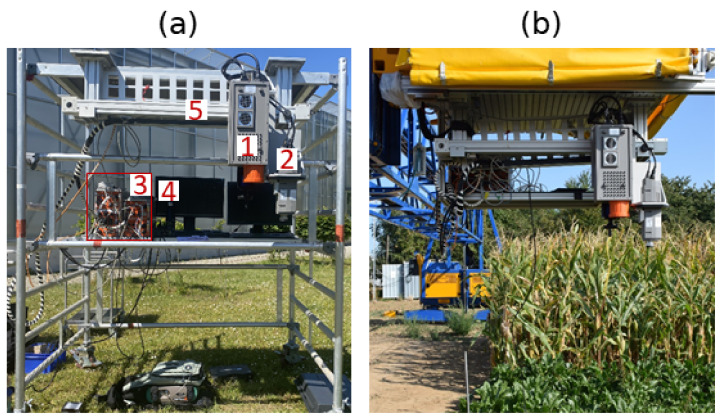
Overview of the HyScreen system: (**a**) HyScreen mounted on a movable scaffolding with components noted in numbers. Legend: 1. fluorescence sensor (FLUO), 2. visible and near-infrared sensor (VNIR), 3. power and control units (PCUs) and data acquisition computers (DACs) of the two sensors, 4. displays, and 5. linear axis. (**b**) HyScreen mounted on the mobile field phenotyping platform.

The HyScreen system consists of two push-broom imaging spectrometers: the fluorescence sensor (FLUO) and the visible and near-infrared sensor (VNIR). This system was built and developed by Forschungszentrum Jülich in cooperation with SPECIM (Spectral Imaging Ltd., Oulu, Finland) as part of the German Plant Phenotyping Network (DPPN). Figure 1a shows the detailed components of the system. Both cameras are mounted side-by-side on a scanning bar to create an overlapping field of view (FOV) and synchronous movement. The system has a main, compact power and control unit (PCU), which is connected to both the VNIR and FLUO sensors. In addition, each sensor has its own PCU and corresponding data acquisition computer (DAC), which includes data acquisition software. With this setup, the HyScreen system can be mounted on a scaffolding (Figure 1a) or a mobile platform in the field (Figure 1b).

The main components of HyScreen are the imaging spectrometers. The VNIR module has a high-speed complementary metal-oxide semiconductor (CMOS) sensor. It covers the spectral range from 400 nm to 1000 nm with a mean spectral sampling interval of 0.78 nm and a mean full width at half maximum (FWHM) of 3.21 nm. The FLUO module has a scientific CMOS (sCMOS) detector. It covers the spectral range from 670 nm to 780 nm with a mean spectral sampling interval of 0.055 nm and a mean FWHM of 0.31 nm. The detailed characteristics of the two spectrometers are shown in Table 1.

### 2.2. Measurement Protocol

HyScreen can be used from two different measurement platforms: it can be installed (i) on a scaffolding at a height of 1.4 m of FLUO and a fixed height of 1.2 m of VNIR above ground (Figure 1a) or (ii) on a mobile gantry system for phenotyping, where the distance between the sensors and the measurement object is adjustable from 1 m up to 3 m above ground (Figure 1b). During measurement, the geometry of the sun, target and sensor have to be considered to avoid shadows from the platform being cast on targets. The sensors are mounted in nadir position and leveled. Spatial and spectral binning, frame rate, integration time, scanning speed, dark-current measurements and measurement range are controlled by the manufacturer’s proprietary software. Two sensors move simultaneously from the beginning of the linear axis to any point on the axis using a motor. Two images from FLUO and VNIR with a shared field of view can be produced by scanning lines and moving the sensors along the target. During data acquisition, the signal level is monitored by a live view that can also display saturated pixels.

Table 1 shows the standard measurement parameters, including the spatial and spectral binning options. To improve the SNR, the spatial and spectral binnings of the VNIR module are set to 2, while the binning of the FLUO module is set to 4 in the spatial dimension and to 2 in the spectral dimension. When measuring at a distance of 1 m between targets and sensors, the spatial resolutions of the FLUO and VNIR pixels are 1.53 mm and 0.89 mm, respectively. The frame rates of the VNIR and FLUO modules are 20 and 10 frames per second (fps), with maximum integration times of 50 and 100 ms, respectively. To acquire square pixels, the scanning speed is determined by the sensor–target distance and the acquisition frame rate. To focus the sensors, we use a sheet with black and white stripes before measurement whenever adjusting the height of sensors. Additionally, 100 dark frames are recorded by closing the electro-mechanical shutter before each measurement. The average dark current is later subtracted from the raw data.

Solar downwelling radiance of HyScreen is derived by measuring calibrated Lambertian diffuse reflectance reference panels made from Zenith Polymer^®^ (SphereOptics GmbH, Herrsching, Germany) placed at the beginning of the scanning area. The reflectance of the panels is chosen to be less than 50% to obtain the highest possible SNR of vegetation objects. During data acquisition, the reference panels should be horizontally leveled in the principal plane and kept free from shadows.

### 2.3. Case Studies

#### 2.3.1. SNR and NER of Reflectance Panels

In order to determine the SNR and corresponding noise-equivalent-radiance (NER) of HyScreen’s FLUO module, Lambertian reference panels with different reflectances were recorded. We chose four 0.2 × 0.05 m panels with reflectances of around 5%, 20%, 50% and 95%. The scene was captured at Forschungszentrum Jülich, Jülich, Germany (50.9097° N, 6.41279° W) on 23 April 2021 at 13:13 Coordinated Universal Time (UTC). ROIs with an average size of 685 pixels were generated to calculate SNR and NER. Since SNR and NER are slightly influenced by the across-track pixel position, the results were calculated as the mean of SNR and NER of the across-track samples.

#### 2.3.2. Experiment with Vegetation and Non-Vegetation Objects

**Figure 2:**
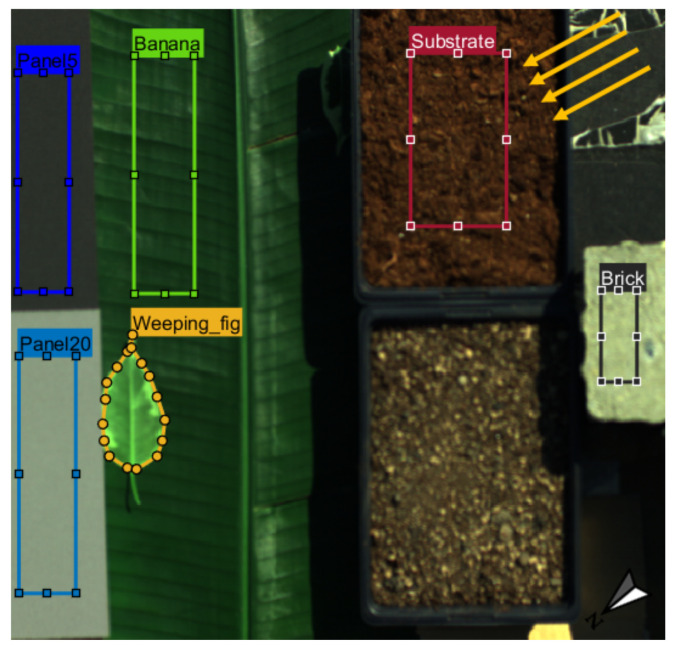
Vegetation and non-vegetation target ROIs used for the HyScreen case study. The 5 and 20% reflectance panels were used for measuring downwelling radiance and empirical line correction. The other targets were used to test the performance of HyScreen: banana leaf, weeping fig leaf, substrate, and brick. The scanning direction along the track was from left-to-right, while spatial pixels across the track are in the vertical direction. The sun zenith angle and azimuth angles were 43.35 and 192.12°, respectively, (yellow arrows). The arrow in the bottom right corner is pointing north.

To demonstrate the performance of HyScreen over various vegetation and non-vegetation targets, one scene including several objects was captured at Forschungszentrum Jülich, Jülich, Germany (50.9097° N, 6.41279° W) on 1 April 2020 at 12:13 Coordinated Universal Time (UTC) with sun zenith angle of 43.35° and sun azimuth angle of 192.12°. On this day, the sun’s zenith angle at solar noon (11:38 UTC) was 43.93°. HyScreen was mounted on the scaffolding shown in Figure 1a and placed on a flat lawn area facing southeast with azimuth angle of 130°, avoiding shadows from surrounding objects. The targets were placed on a leveled, black plastic tray. Two reference panels with 5 and 20% reflectance, one big banana leaf, a sunlit weeping fig leaf, one pot of substrate, and a brick were placed in the scene as ROIs, as shown in Figure 2. The scanning direction of the sensors was from left-to-right in this image, so the light came from the upper right with an angle around 62°. The SNR and NER of the ROIs of two panels were calculated for demonstrating the measurement quality of this scene, and the results are demonstrated in Appendix A.

Standard measurement parameters, as shown in Table 1, were used during the scan. Only the height of the sensors above the targets was slightly different compared to the default settings. The FLUO and VNIR module were mounted at heights of 1.4 m and 1.2 m above the targets, respectively. The integration times were adjusted to the illumination conditions on the measurement day. Data were processed according to the processing chain described in Section 2.4. Regions of interest (ROIs) of the targets were extracted and processed in MATLAB 2021a (The MathWorks, Inc., Natick, MA, USA) [27]. The SNRs and NERs of the ROIs of the panels were calculated by the method in Section 2.4.5 using Python 3.8 [28]. To apply a correction with the empirical line method (ELM) Section 2.4.2, the panels with 5 and 20% reflectance were used to determine the linear relationship between reflectance and at-sensor radiance $L_{\mathrm{at}-\mathrm{sensor}}^{↑}$.

### 2.4. Image Processing Chain

**Figure 3:**
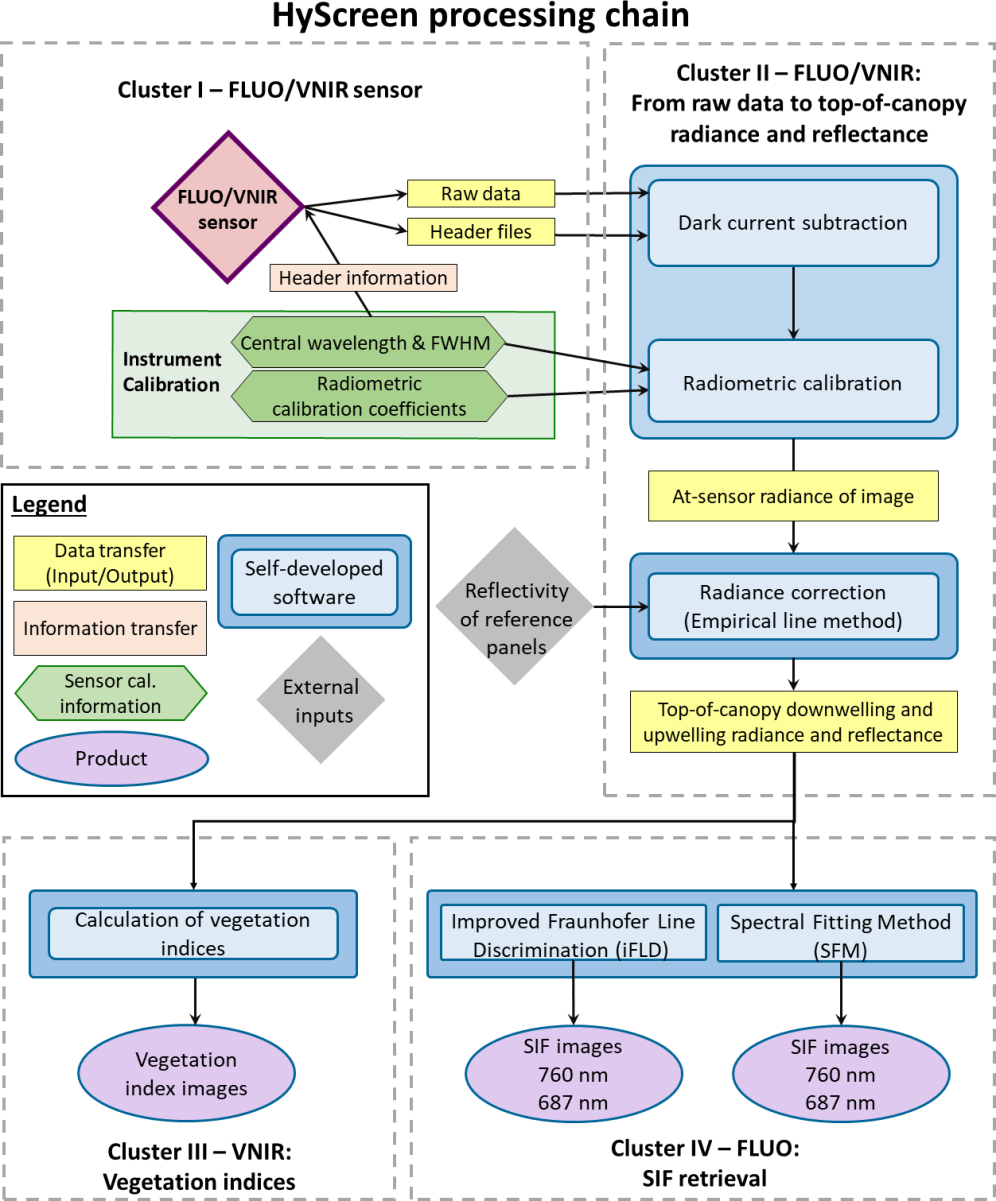
Flowchart of the HyScreen processing chain consisting of the fluorescence sensor (FLUO) and the visible and near-infrared sensor (VNIR) modules divided into four clusters: (I) raw data preparation, (II) from raw data to top-of-canopy downwelling and upwelling radiance and apparent reflectance, (III) vegetation indices and (IV) solar-induced chlorophyll fluorescence (SIF) retrieval. Spatial resolution is represented by full width at half maximum (FWHM).

The HyScreen data processing chain consists of four clusters, as shown in Figure 3. The first cluster summarizes how spectral and radiometric calibration files complement the raw data consisting of the hyperspectral data cubes and header files. The second cluster describes the transfer of raw hyperspectral image cubes recorded by the VNIR and FLUO modules to TOC downwelling radiance, upwelling radiance and reflectance. Self-developed software using MATLAB 2021a [27] is used for dark-current subtraction, radiometric calibration and Empirical Line Method (ELM) correction [29], converting at-sensor radiance into TOC radiance and reflectance values as seen in Section 2.4.1 and Section 2.4.2. The third cluster calculates vegetation indices from data recorded by the VNIR module Section 2.4.3, while the fourth cluster includes SIF retrieval at 687 and 760 nm based on the two methods: improved Fraunhofer line discrimination (iFLD) and the spectral fitting method (SFM) Section 2.4.4.

#### 2.4.1. Raw Data to At-Sensor Radiance

To convert digital numbers from raw data to at-sensor radiance, dark-current frames are averaged and then subtracted from the raw data, normalized by integration time and multiplied by radiometric calibration coefficients. This step is described in Equation (1),

(1) $$L_{\mathrm{at-sensor}}^{↑}\left(\lambda \right)=\frac{{Raw}_{\mathrm{DN}}(\lambda )-{Raw}_{\mathrm{DC}}(\lambda )}{IT}{coeff}_{\mathrm{rad}}\left(\lambda \right),$$

where the ↑ stands for upwelling signals, ${Raw}_{\mathrm{DN}}$ are digital numbers of the raw data cube, ${Raw}_{\mathrm{DC}}$ is dark current, IT (ms) is integration time, ${coeff}_{\mathrm{rad}}$ are radiometric coefficients, $L_{\mathrm{at-sensor}}^{↑}$ is at-sensor radiance in the unit of mW m${}^{-2}$ sr${}^{-1}$ nm${}^{-1}$, and λ indicates the corresponding wavelength. The radiometric calibration coefficients provided by the sensor manufacturer are pixel- and wavelength-dependent.

#### 2.4.2. Empirical Line Method for Radiance and Apparent Reflectance

The sensor radiometric and spectral calibrations as well as the optical characterization (i.e., non-linearity and point-spread function) uncertainties introduce distortion between the at-sensor radiance and the TOC radiance. Here, we call the difference ’offset radiance’. Darker targets and the oxygen absorption bands suffer more from this offset than brighter targets and bands outside the absorption features because the ratio of offset-to-radiance, i.e., the SNR, is relatively higher [30,31].

In this study, we apply the empirical line method (ELM) to convert at-sensor radiance ($L_{\mathrm{at-sensor}}^{↑}$) to TOC radiance ($L_{\mathrm{TOC}}^{↑}$), and here, we call it ’radiance correction’. At least two reference panels with known reflectance (*R*) have to be used to establish a linear relationship to the at-sensor radiance from HyScreen. Calibration of these reference panels has to be done in the laboratory. In the at-sensor radiance image, ROIs covering the reference panels are selected to determine their averages and standard deviations. Based on the known reflectance and the measured at-sensor radiance of the reference panels, a linear relationship can be determined for each wavelength. The intercept on the y-axis showing at-sensor radiance indicates the offset ($L_{\mathrm{offset}}^{↑}$) caused by the different artifacts mentioned above in the measurements. This offset has to be subtracted from at-sensor radiance to obtain TOC radiance, which makes the fitting line run through the axis origin. TOC downwelling radiance ($L_{\mathrm{TOC}}^{↑}$) can then be described as TOC upwelling radiance ($L_{\mathrm{TOC}}^{↑}$) when reflectance equals one. Finally, apparent reflectance ($R_{\mathrm{app}}$) can be calculated according to Equation (2).

(2) $$R_{\mathrm{app}}\left(\lambda \right)=\frac{L_{\mathrm{TOC}}^{↑}\left(\lambda \right)}{L_{\mathrm{TOC}}^{↓}\left(\lambda \right)}.$$

**Figure 4:**
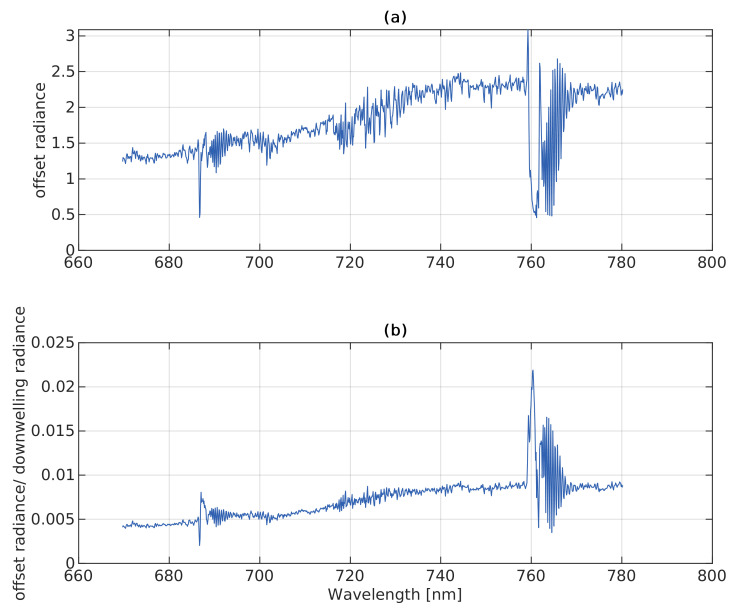
(**a**) Offset of at-sensor upwelling radiance across the FLUO module’s spectral range calculated with the empirical line method (ELM) described in Section 2.4.2. Each value corresponds to the intercept from the linear equation fitted to the 5 and 20% reference panels. (**b**) ratio of offset radiance to downwelling radiance.

In Figure 4a, we can observe the upwelling radiance offset from 670 to 780 nm derived with the ELM from the 5% and 20% panels in Figure 2. Due to the low downwelling radiance in the oxygen absorption features, the offsets within the $O_{2}A$ (1 mW m${}^{-2}$ sr${}^{-1}$ nm${}^{-1}$) and $O_{2}B$ (1.5 mW m${}^{-2}$ sr${}^{-1}$ nm${}^{-1}$) bands are distinctly smaller in comparison to the wavelengths located on the shoulders of both absorption features. Consequently, the ratio of offset-to-downwelling radiance differs within and outside the absorption wavebands, which leads to an infilling feature similar to SIF, as shown in Figure 4b. We can observe that the infilling at $O_{2}A$ is larger than at $O_{2}B$. The offset can lead to around 0.50 and 0.36 mW m${}^{-2}$ sr${}^{-1}$ nm${}^{-1}$ error in SIF retrieval, respectively. Thus, considering the current optical characterization of the system, the ELM correction of the offset radiance is significant for SIF retrieval.

#### 2.4.3. Vegetation Indices

Vegetation indices (VIs) are calculated from TOC reflectance image data collected with the VNIR module. Three indices used in the case study presented in Section 2.3.2 are listed in Table 2: the normalized difference vegetation index (NDVI), which is sensitive to the amount of green vegetation biomass and related to the leaf area index (LAI); the transformed chlorophyll absorption in reflectance index (TCARI), which is inversely correlated to leaf chlorophyll content; and the photochemical reflectance index (PRI), which is an indicator of the state of the xanthophyll cycle and is thus inversely correlated with NPQ.

#### 2.4.4. Solar-Induced Chlorophyll Fluorescence Retrieval

Due to the high spectral resolution of the HyScreen FLUO module, the fluorescence emitted at both $F_{760}$ and $F_{687}$ is retrieved. In this study, the improved Fraunhofer line discrimination (iFLD) method [35] and the spectral fitting method (SFM) [36] were implemented to retrieve SIF from the HyScreen FLUO module. Both methods are widely used by the scientific community for the retrieval of SIF. Detailed descriptions and a comparison of the two methods is provided by [8]. Table 3 summarizes how the iFLD and SFM were implemented in the HyScreem processing chain.

#### 2.4.5. SNR and NER Calculation

As SNR is crucial for assessing the sensor’s suitability to retrieve SIF, in this section, we present the methods used to estimate SNR and NER. For a specific ROI, according to [37], SNR is calculated for each wavelength using Equation (3).

(3) $$\begin{array}{cc} SNR(\lambda ) & =\frac{S(\lambda )}{N(\lambda )}\\ \; & =\frac{\bar{{Raw}_{\mathrm{ROI}}(\lambda )}}{{std}_{\mathrm{ROI}}(\lambda )}\\ \; & =\frac{\bar{{Raw}_{\mathrm{DN}}(\lambda )}-\bar{{Raw}_{\mathrm{DC}}(\lambda )}}{{\left[std^{2}({Raw}_{\mathrm{DN}}\left(\lambda \right))+std^{2}\left({Raw}_{\mathrm{DC}}\left(\lambda \right)\right)\right]}^{1/2}}, \end{array}$$

where *S* and *N* represent signal and noise, respectively, and $\bar{{Raw}_{\mathrm{ROI}}}$ and ${std}_{\mathrm{ROI}}$ stand for the mean and standard deviation of the pixel signals covered by the ROI, respectively. The mean of the pixel signals is calculated from raw data ${Raw}_{\mathrm{DN}}$ from which the dark current ${Raw}_{\mathrm{DC}}$ has additionally been subtracted. The noise corresponds to the standard deviation of the signal and is determined as the square root of the sum of the raw data variance $std^{2}({Raw}_{\mathrm{DN}})$ and dark-current variance $std^{2}\left({Raw}_{\mathrm{DC}}\right)$. When calculating NER, the SNR needs to be set to 1 so that the signal is equal to the noise. With this, the so-called noise-equivalent signal (NES) can be calculated using Equation (4).

(4) $$NES\left(\lambda \right)={\left[std^{2}\left({Raw}_{\mathrm{DN}}\left(\lambda \right)\right)+std^{2}({Raw}_{\mathrm{DC}}\left(\lambda \right))\right]}^{1/2},$$

Finally, the NER can be determined by multiplying the NES with the radiometric calibration coefficients and normalized by integration time (IT), as shown in Equation (5).

(5) $$NER\left(\lambda \right)=\frac{NES(\lambda )}{IT}{coeff}_{\mathrm{rad}}\left(\lambda \right)$$

## 3. Results

### 3.1. Results of SNR and NER of Reflectance Panels

**Figure 5:**
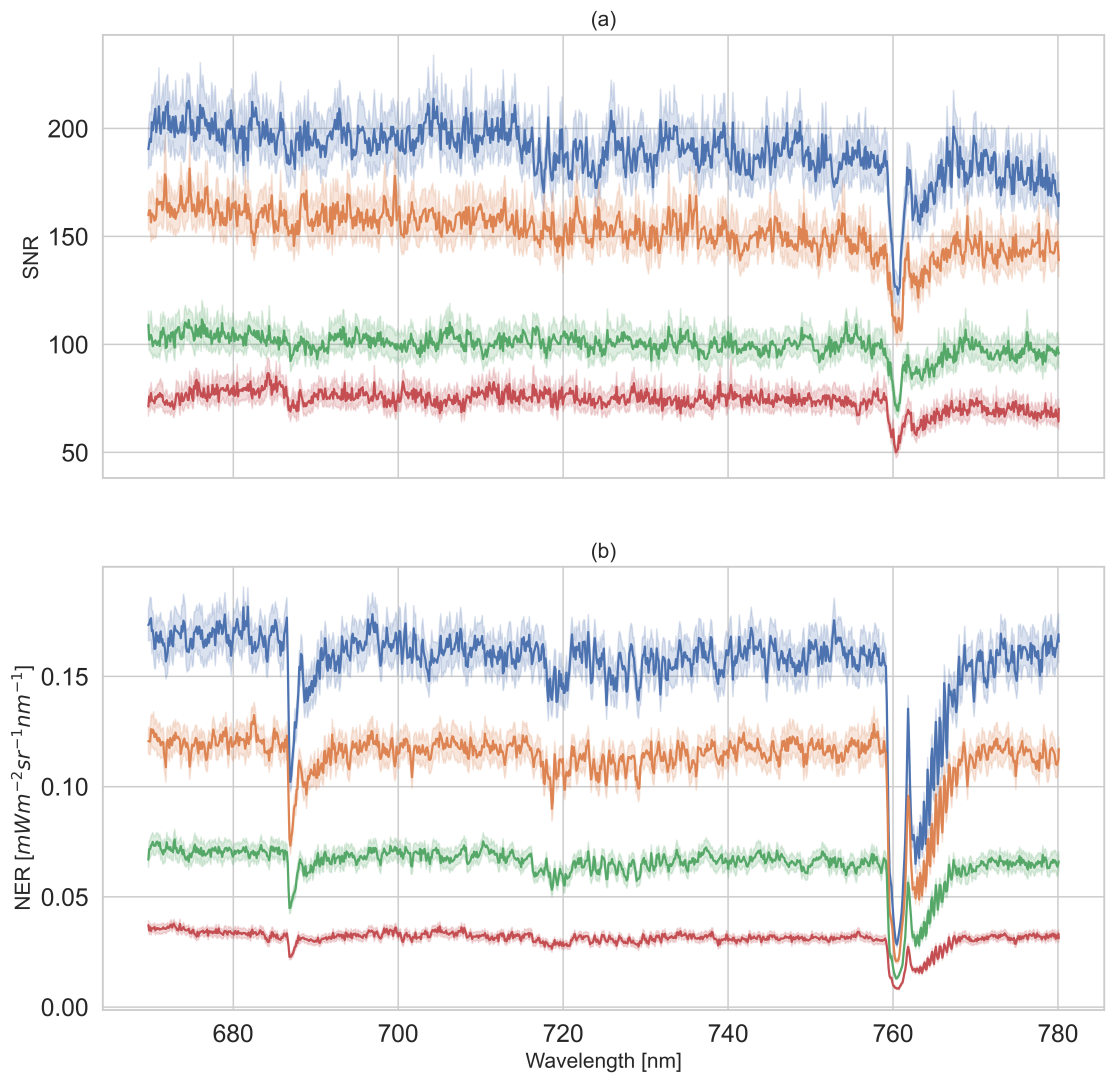
Signal-to-noise ratio (SNR) and noise-equivalent-radiance (NER) of HyScreen’s FLUO module from 670–780 nm derived from Lambertian reference panels with reflectances of 5% (red), 20% (green), 50% (orange) and 95% (blue): (**a**) provides information on the SNR, and (**b**) shows the NERs of four Lambertian reference panels. The solid lines represent mean values of SNR or NER of across-track samples from regions of interest (ROIs), and the light-colored areas illustrate their corresponding standard deviations.

The results of each reference panel are shown in Figure 5. We can observe that the SNR and NER increase with increasing reflectance of panels in Figure 5a,b. For the 5% reference panel, the SNR was close to 75 across wavelengths; regarding the 20%, 50% and 95% panels, the SNR increased up to around 100, 150 and 200, respectively. Since the downwelling radiance within the $O_{2}$ absorption band was much lower compared to the rest of the covered spectral range, the SNR and NER of each reference panel at $O_{2}A$ and $O_{2}B$ were distinctly lower. The reference panels with 95, 50, 20 and 5% had NERs of 0.028, 0.021, 0.013 and 0.008 mW m${}^{-2}$ sr${}^{-1}$ nm${}^{-1}$, respectively, at 760.48 nm, and 0.105, 0.077, 0.048 and 0.023 mW m${}^{-2}$ sr${}^{-1}$ nm${}^{-1}$, respectively, at 687.04 nm. The NERs indicate that darker panels show relatively more noise than brighter panels and thus may introduce higher uncertainty in the SIF retrieval.

### 3.2. Results of the Experiment with Vegetation and Non-Vegetation Objects

#### 3.2.1. Radiance and Apparent Reflectance Spectra

**Figure 6:**
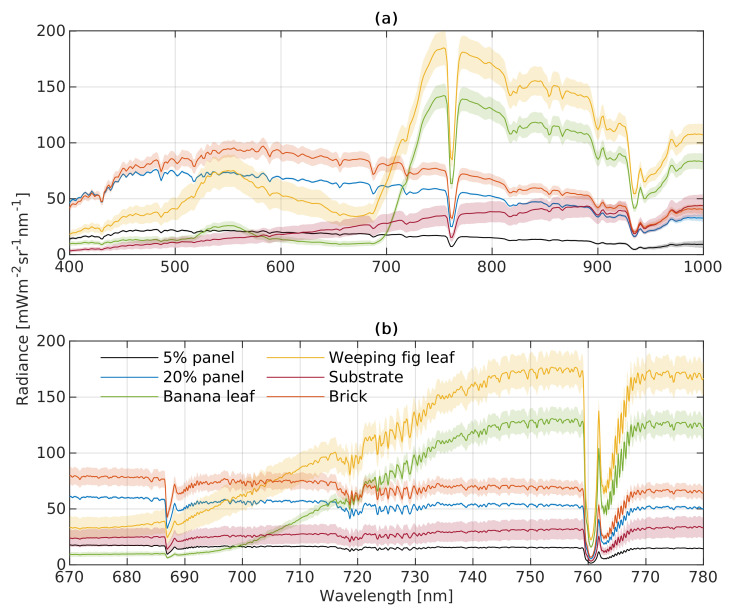
Means and standard deviations of top-of-canopy (TOC) upwelling radiance of different vegetation and artificial targets recorded by the HyScreen (**a**) VNIR and (**b**) FLUO modules. (**a**,**b**) share the same color legend shown in (**b**). The colored lines represent averaged spectra of all pixels of a target covered by an ROI, while the shaded areas represent corresponding standard derivations.

**Figure 7:**
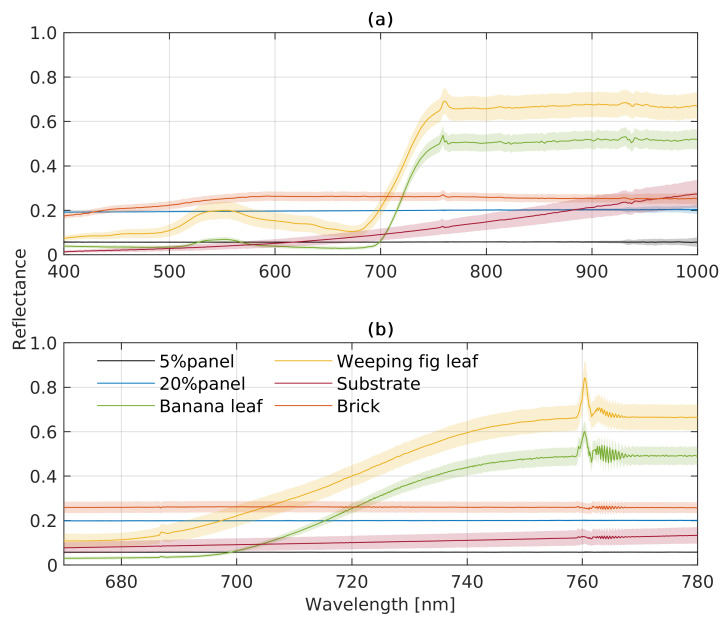
Means and standard deviations of top-of-canopy (TOC) apparent reflectance of different vegetation and artificial targets recorded by the HyScreen (**a**) VNIR and (**b**) FLUO modules. The colored lines represent averaged spectra of all pixels of a target covered by an ROI, while the shaded areas represent corresponding standard derivations.

The FLUO module and VNIR are slightly different but complement each other for vegetation monitoring. Figure 6 shows the radiance spectra and corresponding standard deviations of the ROIs of each target shown in Figure 2. The radiance spectra of the FLUO module (Figure 6b) are slightly lower than those measured with the VNIR module (a). For example, upwelling radiance of the banana leaf at 750 nm recorded with the VNIR module is 12.07 mW m${}^{-2}$ sr${}^{-1}$ nm${}^{-1}$ higher than that of the FLUO module, which corresponds to a 8.93% difference between both modules. In contrast, apparent reflectance is only 1% higher, representing a 2.28% difference, as shown in Figure 7. Meanwhile, VNIR and FLUO both show typical vegetation spectral patterns from banana and weeping fig leave, where green and infrared wavelengths are reflected stronger than those in the red range. The FLUO module has a deeper radiance signature and a sharper apparent reflectance signature than the VNIR module at $O_{2}B$ and $O_{2}A$, which indicates the FLUO module can be used for SIF retrieval and VNIR can be used for vegetation traits retrieval due to its wider wavelength range. Between the ROIs, the banana leaf, weeping fig leaf and substrate are more heterogeneous than the brick and panels, as represented by their standard deviations.

#### 3.2.2. Vegetation Indices and SIF

**Figure 8:**
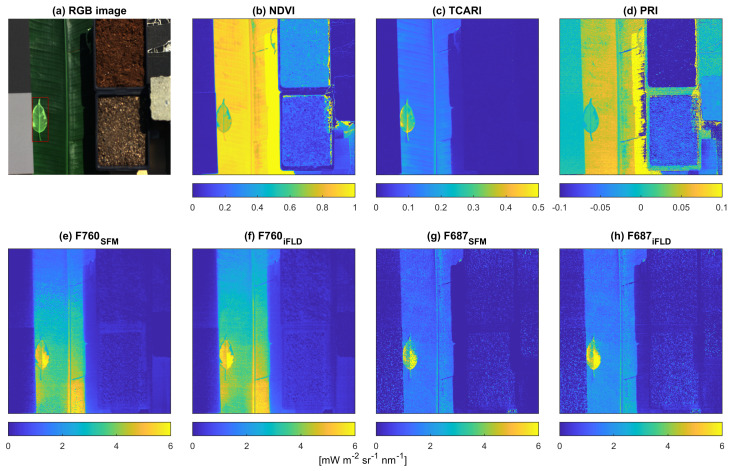
Images of vegetation indices and SIFs at 687 nm and 760 nm: (**a**) true-color composite image of the measured targets; (**b**) normalized difference vegetation index (NDVI), highlighting the vegetation part; (**c**) transformed chlorophyll absorption in reflectance index (TCARI), indicating chlorophyll concentration; (**d**) photochemical reflectance index (PRI) image related to xanthophyll cycle of the NPQ process; (**e**–**h**) retrieved SIF images at 760 nm and 687 nm, respectively, from SFM and iFLD. The ROI of the weeping fig leaf is labeled by a red rectangle.

Figure 8 shows a true-color composite and VI images calculated according to the equations in Table 2 as well as SIF images retrieved based on the two methods described in Section 2.4.4. The means and standard deviations of VIs and SIFs are shown in Table 4 and Table 5. The composition of the scene and spatial variations can be observed from both VI and SIF images. Observing the patterns within ROIs from vegetation targets, NDVI and PRI decrease from the banana leaf to the green area of the weeping fig and to the chlorophyll-deficient area of the weeping fig, which is inverse to the pattern of TCARI. In the NDVI image, vegetation, including sunlit and shaded parts, can be distinguished easily, but TCARI, PRI and SIF show values clearly different between sunlit and shaded parts. The NDVI image (b) shows a clear separation between vegetation (>0.4) and non-vegetation targets (<0.4). Most non-vegetation areas, such as the brick, have averaged NDVI values around 0. Only the shaded areas at the edges of the targets exhibit some artifacts. The substrate consisting of peat shows higher average values (0.32) and standard deviation (0.04) compared to the brick (−0.01 and 0.01, respectively), which is probably due to the remaining plant material reflecting relatively more in the near-infrared than red wavelength [38] and its spatial heterogeneity. In the TCARI image (c), we can distinguish vegetation and non-vegetation easily by a threshold of 0.07, except for the shaded area of the banana leaf, where TCARI has an average value of 0.01. In the PRI image (d), only the vegetation targets typically show reliable PRI values with meaningful information related to NPQ. Interestingly, all three vegetation indices and SIF show differences within the weeping fig leaf according to the differing chlorophyll concentration.

Table 5 shows means and standard deviations of the SIF images calculated for the different ROIs. Results from the SFM method are consistent with those of the iFLD method, showing similar means and standard deviations of SIFs for each of the ROIs. Regarding the mean values of $F_{687}$ and $F_{760}$, SFM has relatively lower values from vegetation targets and values closer to zero from non-vegetation targets compared to the iFLD method, but SFM also seems more heterogeneous than iFLD. For all ROIs, $F_{760}$ is higher and less noisy than $F_{687}$, except for the banana leaf, where std of $F_{760}$ is higher than for $F_{687}$. The patterns of images of $F_{687}$ and $F_{760}$ are different from each other, and both differ from the pattern observed in the vegetation indices. The banana leaf has lower $F_{760}$ than the green area of the weeping fig but higher $F_{760}$ than the chlorophyll-deficient area. However, the banana leaf has the lowest $F_{687}$ compared to the entire area of the weeping fig leaf. Looking at the SIF images, the banana leaf $F_{760}$ shows a stronger spatial variation across the leaf compared to $F_{687}$. Using the SFM result images as examples, the bottom-right part of the banana leaf shows clearly higher $F_{760}$ than other parts, which is consistent with the specular reflection visible in the RGB image. Sunlit parts have higher values than shaded parts. The shaded part of the banana leaf shows $F_{687}$ and $F_{760}$ of 0.1 and 0.67 mW m${}^{-2}$ sr${}^{-1}$ nm${}^{-1}$, respectively, which are much lower compared to the sunlit part of the leaf (1.13 and 1.96 mW m${}^{-2}$ sr${}^{-1}$ nm${}^{-1}$, respectively). The weeping fig leaf has the highest SIF values and standard deviations compared to other ROIs due to its uneven distribution of chlorophyll. The weeping fig leaf with differing chlorophyll content can be distinguished both in $F_{687}$ and $F_{760}$. Although non-vegetation targets should have zero SIF, the results are negative for $F_{687}$ and close to zero for $F_{760}$.

## 4. Discussion

In this manuscript, HyScreen, the first imaging system capable of retrieving fluorescence in the red and far-red at ground level is introduced to the scientific community. In this section, we first evaluate the suitability of Hyscreen to accurately retrieve SIF based on SNR and NER system characterization. Secondly, based on the results of this study, HyScreen optical and radiometric characterization and data processing are discussed. Finally, the retrieved SIF and vegetation trait spatial patterns observed in this study are interpreted, showing the potential of Hyscreen to close the gap between canopy and leaf-level measurements.

### 4.1. SNR and NER Characterization

For SIF measurements, the signal-to-noise ratio (SNR) and spectral resolution are the most important parameters. They can even account for up to 40% error of the SIF retrieval accuracy [39]. The NER results from Section 3.1 indicated that darker panels show relatively more noise than brighter panels and thus may introduce higher uncertainty in the SIF retrieval. Considering a typical vegetation target with 50% reflectance at $O_{2}A$, the relative error of SIF caused by noise would have a range from 0.42% to 1.05% when true SIF has values between 2 and 5 mW m${}^{-2}$ sr${}^{-1}$ nm${}^{-1}$. For vegetation with only 5% reflectance at $O_{2}B$, the relative error would be in the range from 0.46 to 1.15% when $F_{687}$ is in the range of 2 to 5 mW m${}^{-2}$ sr${}^{-1}$ nm${}^{-1}$. The results are consistent with the definition of SNR, where SNR depends on signal amplitude. For most modern spectroradiometers, the majority of noise is photon noise, which is caused by the inherent random number of captured photons forming a Poisson distribution. The higher the expected hits of photons within the integration time, the higher the ratio of the expected value to the standard deviation of hits is, and thus the higher SNR is [30]. Thus, to optimize SNR, the key is to increase signal and reduce noise. Firstly, measurements should be carried out under high intensity of illumination, such as clear sky conditions and sun zenith angles lower than 60° [40]. In addition, spatial and spectral binning reduces noise by averaging values of several pixels [21]. Moreover, the integration time has to be optimized to cover 3/4 of the sensors’ dynamic ranges to achieve an optimal SNR [11]. According to the knowledge above, we recommend using reference panels with reflectance not higher than that of the vegetation; thus, the SNR from the vegetation can be optimized by improving integration time without saturation from non-vegetation targets.

### 4.2. Processing Chain Improvements

Based on the results from the case study, we identified some possible improvements for data collection as well as additional correction of sensor characteristics that could be included in the processing chain. When observing the image of $F_{760}$, we see a vertical heterogeneous distribution of SIF on the banana leaf (Figure 8). In the current processing chain, the downwelling radiance is interpolated from the radiance of ROIs of two reference panels by ELM. In the future, the ELM correction should be spatial pixel-dependent using at least two reference panels covering the entire swath across track.

In addition, the ELM approach is based on the assumption that reflectance is linearly related to upwelling radiance. Non-linearity is a known effect in spectroradiometers: meaning an increasing number of photons does not mean the same proportional increase of digital numbers captured by the detector [37,41]. However, non-linearity is strongest at the low and high ends of the dynamic range of the detector. In the measurement protocol, the integration time is optimized for the scene. Therefore, non-linearity effects should be minimized. Nevertheless, non-linearity correction will be implemented in the processing chain. Further improvement could be the implementation of a correction of stray light in the sensor, described as the point spread function (PSF), such as has been suggested by Albert et al. [42] using a monochromatic laser or Scharr et al. [43] using a double monochromator.

### 4.3. Spatial Distribution of Vegetation Indices and SIF

HyScreen provides us great opportunities for studying SIF distribution originating from biophysical (e.g., leaf structure and optical) and biochemical properties (pigments) of plants. Chlorophyll content and photosynthetic efficiency may explain the variation of SIF across vegetation ROIs. Within the weeping fig leaf, the patterns of $F_{687}$ and $F_{760}$ are quite similar, but $F_{687}$ is slightly higher than $F_{760}$ in the chlorophyll-deficient area. We hypothesize that the lack of linear electron transport from the chlorophyll-deficient area may result in low PSII efficiency and thus high $F_{687}$ [44,45]. However, further analysis is needed to support this hypothesis, which is out of the scope of this study. Taking the banana leaf into consideration, its lower $F_{687}$ compared to that of the weeping fig leaf is probably caused by its high chlorophyll content, indicated by TCARI, and consequently, stronger re-absorption of $F_{687}$. The lower $F_{760}$ from the banana leaf compared to the green area of the weeping fig may be due to higher photosynthetic efficiency or because the weeping fig leaf is lying on top of the banana leaf, which may cause some added background signal.

$F_{760}$ suffers more strongly from directional scattering than F687 [13]. Furthermore, the effect of the bidirectional reflectance distribution function (BRDF) of the target (leaf) is visible in F760 (Figure 8). This effect is combined with the slightly changing sensor viewing direction, which is nadir in the center of the scanning line and 16° at the edges of each scanned line, according to the FLUO sensor’s field of view (Table 1). The combination of sun zenith and azimuth angles (Figure 2) and sensor viewing direction likely explain the heterogeneous nature of $F_{760}$.

With the very detailed spatial information of SIF and vegetation indices, it is possible to investigate how illumination, plant structure and plant physiology interact. For example, it will be interesting to investigate how efficiently plants absorb diffuse light for photosynthesis and how SIF changes accordingly. In this case study, the shaded vegetation parts have lower SIF than the sunlit parts when assuming the same downwelling radiance calculated from sunlit reference panels. The SIF in shaded areas was not further analyzed due to their unknown downwelling radiance and relatively lower SNR compared to the sunlit areas of the scene. However, this very high spatial resolution information is a clear advantage compared to point spectrometers. It enables investigating the ratio of sunlit-to-shaded areas in a scene, and thus, we can correct or remove the shaded parts when calculating SIF from the whole scene.

HyScreen is unique for its high spatial and spectral resolution, capturing both red and far-red SIF and vegetation trait distributions within a scene. Firstly, HyScreen can provide valuable ground measurements that can be used for parameterizing advanced radiative transfer models such as DART [46] and SCOPE [47] for researching the interaction among illumination, canopy structure and viewing angle. Pinto et al. [21] did the pioneering work investigating the leaf angle and orientation effects on $F_{760}$ of sunlit leaves of sugar beets. With HyScreen, we can even go further by evaluating the effects of varying APAR and re-absorption of SIF when combining measurements of canopy structure. The combination of $F_{687}$ and $F_{760}$ is unique for studying the relationship between SIF and photosynthesis, as demonstrated by the work on Arabidopsis by Acebron et al. [48]. With the help of the spatial distribution of SIF retrieved from HyScreen, there is the chance to separate the effects on SIF from leaf angles, leaf orientations and leaf age from physiological effects. Even the energy distribution between PSI and PSII can be investigated [4,49].

## 5. Conclusions

This study aimed to introduce the advanced imaging spectrometer HyScreen for SIF retrieval and demonstrated the measurement protocol and processing chain of both red and far-red SIF. We analyzed the spatial distribution of SIF of simple vegetation targets with differing biochemical properties (chlorophyll content). Uncertainties of SIF from noise, illumination and sensor optics were discussed, and potential corresponding solutions were provided. This study established a framework for SIF retrieval with millimeter-level spatial resolution for the first time for both red and far-red SIF. HyScreen is a valuable addition to proximal sensing of SIF, which can, together with other sensors, be used for investigating SIF propagation from leaf and canopy and the energy distribution between photosynthesises, SIF and NPQ as well as exploiting PRI dynamics.

## Figures and Tables

**Table 1:** Characteristics of the fluorescence sensor (FLUO) and the visible and near-infrared sensor (VNIR) imaging spectrometers of HyScreen. The VNIR module measures in the visible and near-infrared spectral range, while the FLUO module, with its very-fine spectral resolution, only covers the visible red and near-infrared spectral range and was specifically designed to retrieve SIF. FWHM stands for full width at half maximum.

Sensor	VNIR	FLUO
Sensor type	CMOS	sCMOS ${}^{1}$
Dynamic range (bit)	12	14
Spectral range (nm)	400.00–1000.00	669.68–780.22
Mean spectral sampling interval (nm)	0.79	0.06
Mean spectral resolution (FWHM) (nm)	3.21	0.31
Field of view (FOV) (°)	32.71	32.24
Spatial pixels	1312	1512
Standard measurement setup ${}^{2}$		
Spectral binning	2	2
Spectral sampling interval (nm)	1.46–1.61	0.10–0.12
Spectral resolution (FWHM) (nm)	2.42–4.35	0.36 at $O_{2}B$/0.40 at $O_{2}A$
Number of bands	384	1004
Spatial binning	2	4
Spatial pixels	656	378
Swath width of the sensor mounted 1 m above canopy (mm)	587	578
Frame rate (fps)	20	10
Exposure time range (ms)	0.1–50	0.1–100
Power consumption (W)	80	115
Input voltage (V)	12	12

^1^ sCMOS: scientific CMOS chip–chip technology that combines high signal-to-noise ratio, wide dynamic range and fast frame rates with linear sensitivity. ^2^ To improve the SNR, the spatial and spectral binning of the VNIR module are set to 2, while the binning of the FLUO module is set to 4 in the spatial dimension and 2 in the spectral dimension.

**Table 2:** Vegetation indices calculated from TOC reflectance data recorded with the HyScreen VNIR module: normalized difference vegetation index (NDVI), transformed chlorophyll absorption in reflectance index (TCARI) and photochemical reflectance index (PRI). All indices are calculated from averaged reflectance (R) values of small spectral windows located around a central wavelength (nm), which is stated in subscript.

Index	Equation	Reference
NDVI	$\frac{R_{802\pm 4}-R_{672\pm 4}}{R_{802\pm 4}+R_{672\pm 4}}$	[32]
TCARI	$3\left[\left(R_{700\pm 4}-R_{670\pm 4}\right)-0.2\left(R_{700\pm 4}-R_{550\pm 4}\right)\right]\frac{R_{700\pm 4}}{R_{670\pm 4}}$	[33]
PRI	$\frac{R_{531\pm 2.5}-R_{570\pm 2.5}}{R_{531\pm 2.5}+R_{570\pm 2.5}}$	[34]

**Table 3:** Description of the parameters, wavelength ranges, wavelength intervals (WI) and interpolation/model functions used in this study for the iFLD and SFM retrieval methods. Downwelling radiance ($L^{↓}$), reflectance (*R*), fluorescence (*F*), absorption feature (Abs. feature), lower boundary (lb) and upper boundary (ub) are shown in the table. Gaussian function parameters are: *a*, the height of the red and far-red fluorescence curve peaks; *c*, the center of fluorescence peaks; and *b*, the widths of the red and far-red fluorescence spectra.

iFLD						
Method	$L^{↓}$ and *R* Interpolation WI	Abs. Feature WI		Interpolation Method		
*O* ${}_{2}$ *A*	750–780 nm	759.3–768.0 nm		$L^{↓}$: polynomial 2nd grade		
				*R*: linear smoothing spline		
*O* ${}_{2}$ *B*	665–716 nm	683.3–696.9 nm		$L^{↓}$: polynomial 2nd grade		
				*R*: cubic smoothing spline		
SFM						
Method	*F* and *R* Interpolation WI	Abs. Feature WI	Model function	Gaussian function parameters		
				*a*	*c*	*b*
*O* ${}_{2}$ *A*	750–780 nm	759.3–768.0 nm	*F*: Gaussian *R*: Cubic spline	iFLD retrieved fluorescence *ub* = 15, *lb* = 0	740 nm	24
						ub = +Inf
						lb = −Inf
*O* ${}_{2}$ *B*	684–700 nm	686.5–690.0 nm			680 nm	8
						ub = +Inf
						lb = −Inf

**Table 4:** Means and standard deviations of the vegetation indices NDVI, TCARI and PRI for the ROIs of the the different targets shown in Figure 2.

ROIs	NDVI		TCARI		PRI	
	Mean	Std.	Mean	Std.	Mean	Std.
Banana	0.89	0.02	0.12	0.01	0.05	0.01
Weeping fig	0.72	0.05	0.30	0.04	0.01	0.01
Substrate	0.32	0.04	0.00	0.00	−0.13	0.04
Brick	−0.01	0.01	−0.01	0.01	−0.03	0.01

**Table 5:** Means and standard deviations of $F_{687}$ and $F_{760}$ in mW m${}^{-2}$ sr${}^{-1}$ nm${}^{-1}$ derived with the iFLD and SFM SIF retrieval methods for the ROIs of the different targets shown in Figure 2.

ROIs	iFLD687		iFLD760		SFM687		SFM760	
	Mean	Std.	Mean	Std.	Mean	Std.	Mean	Std.
Banana	1.44	0.27	2.59	0.42	1.13	0.26	1.96	0.59
Weeping fig	4.90	1.63	5.17	0.96	4.24	1.76	4.62	0.91
Substrate	−0.14	0.55	0.19	0.15	−0.05	0.43	0.07	0.11
Brick	−0.69	0.95	−0.18	0.16	−0.37	0.74	−0.11	0.14

**Figure A1:**
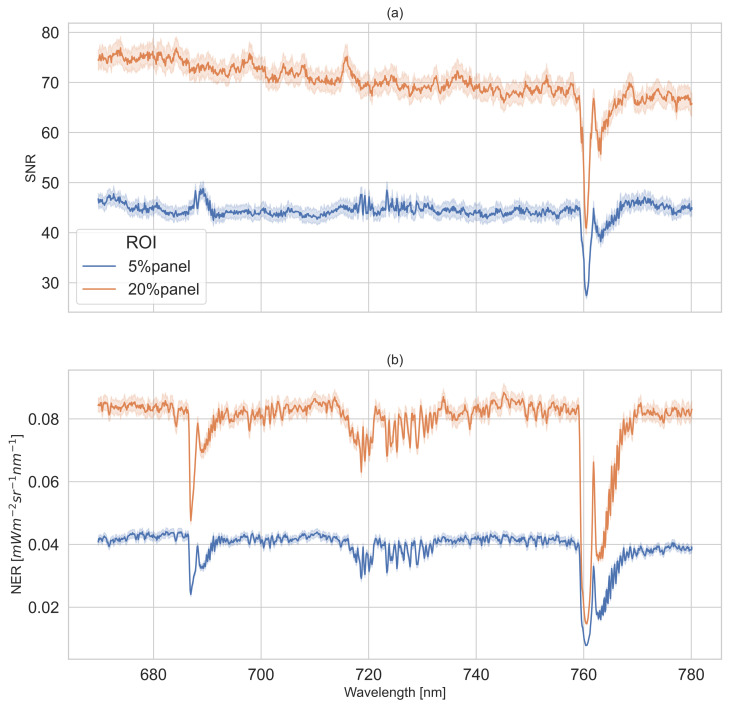
Signal-to-noise ratios (SNRs) and noise-equivalent-radiances (NERs) of HyScreen’s fluorescence sensor (FLUO) module from 670–780 nm derived from Lambertian reference panels with 5% and 20% reflectance: (**a**) provides information on the SNRs, and (**b**) shows the NERs of two Lambertian reference panels. The solid lines represent mean values of SNRs or NERs of across-track samples, which are from regions of interest (ROIs), and the light-colored areas illustrate their corresponding standard deviations.

## Data Availability

Data from this study can be available on request.

## Appendix A. SNR and NER of Two Reference Panels

Figure A1 shows the SNRs and NERs of the two reference panels labeled in Figure 2. At 760.48 nm, the panels with 20 and 5% reflectance had SNRs of 40.90 and 27.44, respectively, and NERs of 0.015 and 0.008 mW m${}^{-2}$ sr${}^{-1}$ nm${}^{-1}$, respectively. At 687.04 nm, 20 and 5% panels had SNRs of 72.44 and 45.21, respectively, and NERs of 0.05 and 0.025, respectively. Despite the NERs being quite similar to those in Section 3.1, the SNRs were lower than that scene, especially at $O_{2}A$.
